# Neurological manifestations of Kawasaki disease and multisystem inflammatory syndrome in children associated with COVID-19: A comparison of two different clinical entities

**DOI:** 10.3389/fped.2022.1088773

**Published:** 2023-01-05

**Authors:** A. Mauro, C. Di Mari, F. Casini, T. Giani, M. Sandini, L. Biondi, V. Calcaterra, G. V. Zuccotti, L. Bernardo

**Affiliations:** ^1^ Pediatric Rheumatology Unit, Department of Childhood and Developmental Medicine, Fatebenefratelli—Sacco Hospital, Milan, Italy; ^2^ Pediatric Department, “Vittore Buzzi” Children’s Hospital, Milan, Italy; ^3^ Department of Pediatrics, Anna Meyer Children’s Hospital, Florence, Italy; ^4^ Department of Internal Medicine, University of Pavia, Pavia, Italy

**Keywords:** Kawasaki disease, multisystem inflammatory disease, neurological involvement, children, COVID-19

## Abstract

Kawasaki disease (KD) is one of the most frequent idiopathic vasculitis in children, affecting medium- and small-sized vessels. Multisystem inflammatory syndrome in children (MIS-C) associated with COVID-19 has recently emerged as a new systemic hyperinflammatory condition affecting children some weeks after an acute COVID-19 infection. KD and MIS-C share different aspects and differ in many others: patients affected by MIS-C are usually older, with prominent gastrointestinal manifestations, diffuse adenopathy, extensive conjunctivitis, myocardial damage, leukopenia, and thrombocytopenia at the laboratory exams. Both conditions can present neurological complications. The aim of this manuscript is to provide a narrative review of neurological involvement in KD and MIS-C. A comprehensive review literature has been performed, and the main clinical features have been analyzed, contributing to neurological differential diagnosis

## 1. Introduction

Kawasaki disease (KD) is an acute inflammatory disease that typically affects medium- and small-sized muscular arteries, particularly the coronary vessels (1). KD is one of the most frequent vasculitis in pediatric patients, with the highest incidence in patients of Asian ancestry (239.6 cases per 100,000 children under 5 years of age) and the lowest incidence in those of European descent (10–15 per 100,000 children under 5 years of age) (2). KD is predominantly, but not exclusively, observed in young children, with most of the cases occurring in children between 6 months and 5 years of age (3).

The exact pathogenesis is still unclear, and a complex interplay of genetic predisposition, immunity, and environmental factors is involved in disease occurrence and outcome (4).

The diagnosis of KD is based on clinical diagnostic criteria and exclusion of other possible causes, as no specific markers are available. Classic KD presents with a fever of at least 5 days of duration associated with ≥4 of the five clinical criteria (cutaneous rash, cervical lymphadenopathy, conjunctivitis, mucositis, extremity changes) (5). Diagnostic criteria are shown in Table 1. When ≥4 of the main features develop early in the disease course, the diagnosis can also be made on the fourth day of fever (3). When a prolonged fever, in the presence of evocative laboratory or echocardiographic findings, is associated with fewer than four clinical criteria, KD is defined as incomplete (3).

**Table 1:** Diagnostic criteria for KD, incomplete Kawasaki disease (IKD), and atypical Kawasaki disease (AKD) (3, 5).

**Diagnostic criteria** **Kawasaki disease:** Fever lasting >5 days without other causes with at least 4/5 of ○ polymorphous rash, ○ oral mucous changes, such as injected or fissured lips or strawberry tongue, ○ cervical lymphadenopathy, ○ bilateral bulbar conjunctival injection, and ○ peripheral extremity changes, including erythema of palms or soles, periungual desquamation, and edema of hands or feet. **Incomplete Kawasaki disease:** ○ Fever without any other reason without a sufficient number of clinical diagnostic criteria, associated or not coronary artery aneurysms. **Atypical Kawasaki disease:** ○ Fever without any other reason that lasts 3–5 days and is associated with symptoms different from classic clinical features associated or not with coronary artery aneurysms.

KD, Kawasaki disease; IKD, incomplete Kawasaki disease; AKD, atypical Kawasaki disease.

In addition to the classic criteria, many different features such as arthritis, uveitis, pancreatitis, abdominal pain, jaundice, and also neurological manifestations can enrich the clinical picture, sometimes misleading the correct diagnosis (3).

Cardiovascular involvement is the most important risk factor for morbidity and mortality in KD patients, both in the acute and chronic phases (5). Up to a quarter of untreated patients develop coronary artery aneurysms (CAAs); with intravenous immunoglobulins (IVIG) treatment, coronary complications reduce to 4%–6% of the patients (6). The only standardized treatment is now represented by a single infusion of IVIG (2 g/kg) in association with acetylsalicylic acid (ASA) at an anti-inflammatory dose (30–50 mg/kg/day) in the acute febrile phase and then shifting to a lower dose (3–5 mg/kg/day) (6). However, 10%–20% of patients are resistant, with a recrudescent or persistent fever at least 36 h after the end of IVIG infusion (4). Different treatment options are available for resistant patients, from a second IVIG dose to pulses of methylprednisolone 30 mg/kg/day (maximum dose: 1 g) for 3 days and biologics such as anti-IL1 and anti-TNF drugs, although it is still uncertain which one of these could represent the best choice (4, 7).

Since April 2020, together with the COVID-19 outbreak, an increased number of children have been identified presenting with a hyperinflammatory condition (8). These patients presented persistent high fever, mucocutaneous involvement (rash, conjunctivitis, swollen and cracked red lips, hand and feet edema), lymphadenopathy, gastrointestinal symptoms (vomiting, abdominal pain, and/or diarrhea) and often developed a cardiac dysfunction (electrocardiogram abnormalities, myocarditis, valvular dysfunction, coronary aneurysm or dilatation, shock) resembling KD and toxic shock syndrome (9).

This new systemic inflammatory condition, following the exposure to SARS-CoV-2, has been formally recognized by the US Centers for Disease Control and Prevention (CDC) (10), the UK Royal College of Paediatrics and Child Health (RCPCh) (11), and the World Health Organization (WHO) (12). Different names have been used to define it, such as multisystem inflammatory syndrome in children (MIS-C) or pediatric inflammatory multisystem syndrome (PIMS/PIMS-TS), and different case definitions have been proposed. Based on similarities with KD, treatments have been derived from the armamentarium of KD drugs. Diagnostic criteria are given in Table 2. First-line treatment for MIS-C is currently represented by a combination of IVIG 2 g/kg and intravenous methylprednisolone 1–2 mg/kg/die. In addition, low-dose ASA (3–5 mg/kg/die) is recommended in all patients without increased bleeding risk. Patients with persistent fever or significant end-organ involvement after 24 h from the initial treatment are considered to have a refractory disease: in such patients, intensification of treatment includes high-dose glucocorticoids (10–30 mg/kg) or anakinra or infliximab. Unlike in KD, a second dose of IVIG is not recommended (13).

**Table 2:** Diagnostic criteria of MIS-C (CDC, WHO, and Royal College) (10–12).

	CDC	WHO	Royal College
	MIS-C	MIS-C	PIMS-TS
Age	<21 years	0–19 years	Children
Fever	Fever >38°C for 3–24 h	Fever 3–72 h	Persistent fever >38.5°C
Clinical features	Laboratory evidence of inflammation and evidence of clinically severe illness requiring hospitalization, with multisystem (≥2) organ involvement (cardiac, renal, respiratory, hematologic, gastrointestinal, dermatologic, or neurological)	Two of the following: - Rash or bilateral nonpurulent conjunctivitis or mucocutaneous inflammation signs (oral, hands, or feet) - Hypotension or shock - Features of myocardial dysfunction, pericarditis, valvulitis, or coronary abnormalities (including ECHO findings or elevated Troponin/NT-proBNP) - Evidence of coagulopathy (by PT, PTT, elevated d-dimers) - Acute gastrointestinal problems (diarrhea, vomiting, or abdominal pain) AND Elevated markers of inflammation	Persistent inflammation and evidence of single or multiorgan dysfunction; this may include children fulfilling full or partial criteria for Kawasaki
COVID-19 infection	Positive for current or recent SARS-CoV-2 infection by RT-PCR, serology, or antigen test or exposure to a suspected or confirmed COVID-19 case within the 4 weeks prior to the onset of symptoms	Evidence of COVID-19 (RT-PCR, antigen test, or serology positive) or likely contact with patients with COVID-19	SARS-CoV-2 PCR testing may be positive or negative
Other	No other plausible diagnoses	No other plausible diagnoses	No other plausible diagnoses

MIS-C, multisystem inflammatory syndrome in children; CDC, Centers for Disease Control and Prevention; WHO, World Health Organization; PT, Prothrombin time; PTT, Partial thromboplastin time.

Involvement of the nervous system in a systemic inflammatory disorder is not unusual (12–14). Recently, similar to reports in multisystemic vasculitis associated with KD, several neurological manifestations have been reported in MIS-C.

This narrative review aims to analyze the neurological involvement in KD and MIS-C to highlight the analogies and differences between these distinct diseases facilitating the diagnosis.

## 2. Methods

This narrative review was performed in accordance with IMRAD (Introduction, Methods, Results, and Discussion) approach (15) and presented a nonsystematic analysis of literature on the neurological manifestations in KD and MIS-C. The authors considered original scientific papers, case reports/series, and reviews of major relevance in the English language published in the past 10 years (2012–2022). PubMed, Scopus, EMBASE, and Web of Science were utilized as electronic databases for this research.

Keywords (alone and/or in combination) were used to select relevant articles, such as, in particular, the following: (“Kawasaki Disease” AND (“Neurological complication” OR “Neurological involvement” OR “Neurological”) AND (“Child” OR “Children” OR “Childhood”); (“Multisystem iperinflammatory disease” AND “SARS CoV-2” OR “Covid-19” OR “Coronavirus 19” AND “Child” OR “Children” OR “Childhood”).

The contributions were independently collected by CDM, FC, and MS and reviewed and discussed by AM, TG, and PB. The resulting draft was critically revised by AM, VC, GVZ, and LB.

The final version was then approved by all.

## 3. Results and discussion

### 3.1. Neurological involvement in Kawasaki disease

Neurological involvement in KD has been described in the literature with an incidence of 1%–30% of patients (16). Pathophysiology of neurological involvement in KD seems to be a consequence of cerebral vasculitis. An inflammatory storm follows the activation of both the adaptive and innate immune response, with the production of matrix metalloproteinases and interleukins (such as IL-17 and IL-6) and deposition of immune complexes (17, 18).

Some genetic variations of human leukocyte antigen (HLA) and polymorphisms of some cytokines and interleukins may influence the susceptibility to develop KD, the immune activation, and the response to IVIG treatment (18).

Neurological symptoms can be variable and poorly specific, like headache, meningism, or irritability. They may appear before the onset of typical KD signs and symptoms, leading to the risk of misdiagnosis, delayed treatment, and consequent increased rate of complications, such as coronary artery lesions (CALs) (19).

#### 3.1.1. Headache

Headache is often associated with KD (20–22). Liu et al. reported an incidence of 16.3% (20), while Yun et al., in their case series of 121 patients, reported an incidence of 2.5% (21). Although headache is a nonspecific symptom, it should be considered in the suspicion of KD neurological involvement (22).

#### 3.1.2. Aseptic meningoencephalitis

Aseptic meningoencephalitis represents the most frequent neurological complication of KD, accounting for about 4%–5% (23).

The etiopathogenesis of aseptic meningoencephalitis is supposed to be linked to vasculitis of arteries, venules, and capillaries. Actually, histopathological reports often revealed signs of cerebral vasculitis with characteristics of periarteritis, endarteritis, and periarterial cuffing (24). The most common symptoms of aseptic meningoencephalitis are irritability and deterioration in responsiveness, which can appear before the development of classical clinical symptoms (25–28).

However, the association with other neurological manifestations has been reported in many case series (19, 27, 28).

The analysis of cerebrospinal fluid may reveal unspecific alterations such as pleocytosis and mildly elevated myelin basic proteins (307 pg/ml). Similarly, radiological findings are not univocal, varying from cerebral infarctions to reversible T2 hyperintensity in the corpus callosum, posterior reversible encephalopathy, and subcortical lesions (29).

Most cases of meningoencephalitis respond to IVIG and acetylsalicylic acid therapy. Nevertheless, Ogata et al. and Stojanovic et al. recommended IVIG retreatment and/or addition of corticosteroids in resistant cases (30, 31).

#### 3.1.3. Subdural effusion

Few cases of KD have been reported to be associated with subdural effusion during the acute phase of the disease; all rapidly resolved after the standard treatment with IVIG and high-dose acetylsalicylic acid treatment (32–34). It seems that concomitant inflammation and damage in dural vessels may diffuse liquids into the subdural space (35).

Aoki reported a case of a 6-month-old girl who presented intracranial hypertension and retinal hemorrhages treated with glycerol and subdural tape. At the 7th month of follow-up, she had no neurological sequelae, and a CT scan confirmed the reduction of subdural fluid collection (32).

Interestingly, an association between subdural effusion and arrhythmias has also been reported.

Bailie et al. described a 6-month-old boy with bilateral subdural collection and prolonged right-sided seizure associated with tachycardia. The patient had an optimal response to IVIG and high-dose aspirin therapy. Follow-up imaging showed complete resolution of the subdural effusion (33).

Chou et al. described a case of a 4-month, 10-day-old boy with anterior fontanelle bulging and moderate bilateral subdural effusion associated with paroxysmal supraventricular tachycardia (about >200 beats/min). Paroxysmal supraventricular tachycardia was treated with adenosine, amiodarone, and propranolol, while subdural effusion had a rapid resolution after IVIG administration (34).

The relationship between subdural effusion and arrhythmias may be linked to the vasculitis damage induced by KD, which affects both dural vessels and canaries, inducing alterations of the cardiac rhythm (32–36).

#### 3.1.4. Cranial nerve palsy

Cranial nerve palsy is a rare complication of KD, most frequently affecting facial and abducens nerves and occasionally oculomotor and glossopharyngeal ones (19, 22, 37–41).

Usually, facial nerve palsy (FNP) shows a self-limiting course lasting from 2 days to 3 months. However, IVIG treatment may reduce the time of recovery (37, 40, 42).

Moreover, it has been described as an association between FNP and an increased risk of coronary arterial involvement (37, 39, 43, 44).

The mechanism underlying FNP may be related to an important vascular inflammation burden associated with a concomitant risk of coronary artery aneurysms.

Kocabaş et al. described an 8-month-old infant with incomplete KD who developed a monolateral FNP and a saccular aneurysm of the right coronary artery. While the FNP responded to the first line of treatment, the coronary involvement worsened (43).

Orgun et al. observed a 4-month-old girl with an incomplete KD developing FNP and a saccular aneurysm in the proximal right coronary artery successfully treated with IVIG (42).

Moreover, the response to IVIG treatment is still controversial.

Yuan and Lu reported two cases of children with fever and unilateral facial droop associated with aneurysms of coronary arteries compatible with incomplete KD. In both cases, the standard treatment was performed with complete remission of both FNP and aneurysms (45, 46).

On the other hand, Yu et al. reported a 7-month-old girl with bilateral FNP debuted 14 days after the first symptoms of KD associated with aneurysms of coronary arteries, and both partially responded to IVIG treatment and aspirin, suggesting a more severe KD course (47).

Abducens nerve palsy (ANP) has been reported in four children (48). Actually, Guven et al. and Warzburger and Avner reported the first cases of ANP, whose etiopathogenesis seems to be related to vasculitis and other cerebral manifestations in KD (49, 50). Moreover, Rodríguez-Lozano et al. reported a case of a 6-year-old boy with incomplete KD treated with IVIG who developed ANP during the 5th day of treatment, suggesting a possible role of IVIG in the pathogenesis of this manifestation (51).

#### 3.1.5. Cerebral vasculitis and stroke

Cerebral vasculitis is a rare and unexpected manifestation in KD.

Vascular inflammation induces diffuse endothelial damage, increasing the risk of microhemorrhages and aneurysm formation and predisposing to a prothrombotic state (52).

Nikkhah reported a 4-year-old boy with an incomplete KD who presented hemiparesis and hemiplegia, exaggerated deep tendon reflexes, and aphasia, in which the neuroimaging study revealed a left middle cerebral artery obliteration (53).

IVIG treatment is supposed to play a potential role in the pathogenesis of the stroke, contributing with different pathophysiologic mechanisms to the thrombotic risk such as vasospasm, increased blood viscosity, erythrocyte aggregation, and platelet activation (54, 55).

Sabatier et al. described an 18-month-old girl with a stroke caused by a complete occlusion of the left internal carotid artery that occurred 1 day after IVIG infusion, on the 10th day of fever (56). The prolonged inflammation and the prothrombotic effect linked to IVIG could be hypnotized to have played a crucial role in this neurological event (56).

Furthermore, large aneurysms and hypokinetic cardiomyopathy, which occasionally complicate the disease course, can lead to thrombus formation and ischemic consequences (57).

Neurological manifestations can be present at the onset of KD, leading to delayed diagnosis, as described in a 15-month-old febrile boy who developed an ischemic stroke and was subsequently diagnosed with coronary abnormalities suggestive of KD (58).

#### 3.1.6. Encephalopathy and seizures

According to the 21st nationwide survey (2009–2010) in Japan, encephalopathy in KD is reported to have an incidence of 0.09% of the cases (59). Takanashi et al. and Yoshihara et al. described several cases of mild encephalopathy with a reversible splenial lesion treated with IVIG and associated with a good prognosis (60, 61).

Febrile seizures are uncommon in the absence of aseptic meningoencephalitis and are limited to case reports (29); actually, the incidence is low compared to ones of the general pediatric population <5 years of age (62, 63).

In a recent retrospective study conducted on Taiwanese children, KD emerged as a possible risk factor for epilepsy, especially in those aged <5 years. The incidence rate of epilepsy resulted in being higher in KD patients with respect to a non-Kawasaki control cohort (47.98 vs. 27.45 every 100,000 person-years), and authors supposed that epilepsy could be the consequence of a parenchymal injury liked to neuroinflammation (64).

#### 3.1.7. Long-term neurodevelopmental outcomes

Neurodevelopmental disorders (NDDs) in pediatrics have a prevalence ranging from 3% to 18%, with a variation among different populations (65–70).

Few studies in the literature have investigated the relationship between KD and possible long-term neurodevelopmental outcomes (71, 72). A recent one, conducted by Lin et al. in 2019, retrospectively studied a cohort of 612 Taiwanese children to investigate the occurrence of NDDs (including autism spectrum disorder, attention-deficit/hyperactivity disorder, sleep-associated disorders, Tourette syndrome, language-communicative disorders, and intellectual disorders) and epilepsy onset after KD. They found epilepsy and Tourette syndrome to have higher rates with respect to both Taiwan and worldwide incidence, whereas no differences were found among other disorders (72, 73).

In another study including 4,597 KD survivors, around 9% of the cases matured developmental disorders mostly after 0–5 years from KD diagnosis, 1% showed intellectual disability, and 0.5% showed attention-deficit/hyperactivity disorder after a prolonged period of observation. Anxiety disorders have been documented in at least a quarter of the children with a recent KD diagnosis (74).

Other studies did not find any differences between children with a history of KD with respect to the general population (75, 76).

Therefore, currently, there is little evidence that KD may be related to long-term neurodevelopmental outcomes, especially because they are often multifactorial disorders that may be related to other genetic and environmental factors.

#### 3.1.8. Sensorineural hearing loss

Sensorineural hearing loss (SNHL) in KD is a well-known KD complication (71, 77). This association seems to be linked to small vessel inflammation; however, ASA may also contribute to causing ototoxicity (77, 78) as well as anemia, thrombocytosis, and delayed initiation of IVIG treatment (after >10 days) (19, 79, 80). Usually, SHNL appears within the first month of the diagnosis, and most cases are transient (77). Sundel et al. described the first five case series, and of them, four had no significant improvement (79). Moreover, Novo et al. reported the case of a boy with classic KD treated with IVIG and aspirin with an optimal response who developed an SNHL after 2 weeks from the disease onset. Thus, after excluding salicylate toxicity, he was treated with steroids and tape with an improvement on the right side but with definitive hearing loss on the left side at the 2 years of follow-up (81).

Kara et al. described a patient with bilateral SHNL, which improved after steroid treatment (82).

However, Robinson et al., in a large retrospective analysis, did not confirm an increased number of cases developing hearing loss in KD patients with respect to the general population (74).

### 3.2. Neurological involvements in MIS-C

Neurological involvement in children with MIS-C is frequently observed, but it is still clinically and epidemiologically not clearly defined. Symptoms such as meningism, headache, drowsiness, irritability, hypotonia, confusion, and severe encephalopathy have been commonly reported (83–86).

Different percentages of neurological involvement are reported in the literature.

In a multicenter study conducted in the United States, 5% of patients were identified to present with severe neurological complications (87). An Italian study reported that in their center in Milan, where each patient admitted for MIS-C was assessed by a pediatric neurologist, 93% of the cases showed a neurological involvement. They identified two different profiles of neurological involvement: in 68% of cases, it was mild and nonspecific, while in 26%, it was moderate-severe; all patients in both categories obtained remission after treatment with steroids and intravenous immunoglobulins (88).

Pathophysiology of neurological involvement in MIS-C seems to be related to cerebral and vascular inflammatory responses with damage of the blood–brain barrier (BBB) and dysregulation in neuronal mediators and receptors, caused by an immunological hyperactivation following SARS-CoV-2 acute infection and by molecular mimicry mechanisms involving SARS-CoV-2 spike protein and neuronal gangliosides. Furthermore, hypoxia, secondary to cardiovascular and pulmonary involvement, can determine neurological manifestations; a state of coagulopathy, secondary to hyperviscosity, venous stasis, vasculitis, and myocardial dysfunction can lead to the development of ischemic stroke (89).

#### 3.2.1. Nonspecific symptoms

Headache represents one of the most reported symptoms, along with fatigue, myalgias, and altered circadian rhythm (88, 90). In their case series of 30 patients, Mihai et al. (90) reported an incidence of headache of 37%, while Bova et al. (88), in their case series of 62 patients, reported an incidence of headache of 68%, followed by asthenia (56%), anorexia (16%), muscular pain (12%), apathy (12%), altered circadian rhythm (4%), and photophobia (4%). These symptoms, even unspecific, have been observed to be associated with other neurological manifestations and should raise the concern of possible neurological involvement (88).

#### 3.2.2. Encephalitis and encephalopathy

Several cases of encephalitis and consequent encephalopathy have been reported, mostly among younger children with MIS-C (median age 5 years). Typical presentation includes drowsiness, irritability, and mood deflection (86, 88, 91–95). In these patients, electroencephalography typically shows a slowing pattern and/or epileptic abnormalities, while MRI often can detect cytotoxic lesions of the corpus callosum (CLOCC) or acute disseminated encephalomyelitis (ADEM)-like lesions (85, 88, 91, 93, 94). CLOCC is generally a reversible condition, which has also been documented in other kinds of encephalopathies, such as viral and inflammatory or because of toxins, nutritional deficiencies, seizures, and in KD (89, 96). Among some patients with ADEM-like lesions, autoantibodies against cerebral molecules have been found (97).

Olivotto et al. described a series of seven patients with a median age of 6 years affected by MIS-C who developed acute encephalopathy. Drowsiness, irritability, and mood deflection were common neurological presentations: three out of seven patients showed milder symptoms, always associated with a headache, while four patients manifested a more severe phenotype with both focal and diffuse neurological signs. EEG showed in all either a diffuse or a mainly posterior slow activity, whose severity and duration were proportional to the severity of the clinical involvement. MRI and cerebrospinal fluid analysis were normal in children with focal signs. All cases were successfully treated with IVIG 2 g/kg and an increase in the dose of methylprednisolone from 2 to 30 mg/kg/day for 3 days (93).

De Paulis et al. analyzed anti-inflammatory and proinflammatory mediators in a child with MIS-C developing encephalopathy, showing increased levels of IL-1B, IL-6, IL-10, and TNFa and decreased levels of IL-1RA and BDNF (a neuroprotective anti-inflammatory cytokine) (92). This inflammatory cascade can explain the good response to the anti-inflammatory treatment (steroids and intravenous immunoglobulins) reported in the described cases, with rapid improvement in clinical symptoms and secondary improvement in EEG patterns (86, 88, 92, 94).

Kashyap et al. described a 7-month-old baby boy with MIS-C who developed a status epilepticus, refractory to first- and second-line antiepileptic therapy. Cerebrospinal fluid examination and MRI were normal. He successfully responded to steroids and IVIG (98).

#### 3.2.3. Cerebellitis

Acute cerebellar involvement has been rarely described as a complication of MIS-C in isolated case reports, mostly in the queue of an acute episode of diffuse transient encephalopathy. The first report, by Akcay et al., described a 3-year-old previously healthy child who developed ataxia, dysarthria, and nystagmus while recovering from an acute encephalopathy 1 week after the initiation of the treatment (cefotaxime, vancomycin, acyclovir, IVIG, high-dose corticosteroids). MRI showed bilateral sign changes in the cerebellar hemisphere, while cerebrospinal fluid analysis was normal. This case of cerebellitis gradually improved on low-dose corticosteroids and aspirin (99). Similarly, Kumar et al. described a case of an 11-year-old boy who developed cerebellar signs (titubation, tremors, dysmetria, ataxia, dysarthria) while recovering from an acute encephalopathy 3 days after starting the anti-inflammatory treatment. MRI and cerebrospinal fluid analysis were normal. He was successfully treated with high-dose corticosteroids (100).

#### 3.2.4. Delirium

Little has been published about the occurrence of neuropsychiatric disorders in patients with MIS-C; case reports of delirium have been described among otherwise healthy adolescents (97, 101). According to the fifth edition of the Diagnostic and Statistical Manual of the American Psychiatric Association (DSM-5), delirium is defined as an acute disturbance in attention and awareness, whose severity fluctuates over the day, and in one additional component of cognition (memory, orientation, language, visuospatial ability, perception), which is a direct consequence of another medical condition/drug intoxication or withdrawal/toxin exposure and cannot be explained neither by a neurocognitive disorder nor by a reduced level of arousal such as in coma (102).

Hutchison et al. reported the case of a previously healthy 14-year-old boy with MIS-C who developed an acute and fluctuating alteration in awareness, attention, memory, and planning, along with psychosis and agitation, who gradually recovered with the improvement of the underlying inflammatory syndrome (101).

#### 3.2.5. Cerebrovascular disease

MIS-C promotes a state of coagulopathy secondary to vasculitis, myocardial dysfunction, venous stasis, and hyperviscosity related to the severe illness. Even if very uncommon in pediatric age, all these factors can lead to the development of cerebrovascular complications (103). Beslow et al. reported the prevalence of 8 cases of acute ischemic stroke out of 971 hospitalized children with SARS-CoV-2 acute infection (0.82%): only 2 out of these 8 patients met the diagnostic criteria for MIS-C (104).

Moreover, Chang et al. and Tiwari et al. described three cases of previously healthy girls (15, 16, and 9 years old, respectively) diagnosed with MIS-C who developed large vessel occlusive acute ischemic stroke. Clinical manifestation consisted of aphasia and hemiparesis in the first patient, sensory obtundation and aphasia in the second patient, and facial and body hemiplegia and reduction in the Glasgow Coma Scale in the third patient. Cerebral CT and CT angiography allowed the diagnosis of ischemic stroke. All these patients were successfully treated with intravenous immunoglobulins, steroids, and anticoagulants (103, 105).

#### 3.2.6. Pseudotumor cerebri

Pseudotumor cerebri, also called idiopathic intracranial hypertension, is the consequence of increased intracranial pressure in the absence of an obvious reason, whose symptomatology can mimic a brain tumor. Some case reports have described the occurrence of this situation in children with MIS-C. The pathophysiologic mechanisms at the basis of this relation are not clear but seem to be related to systemic inflammation: several inflammatory mediators (e.g., TNFa, IL-1, and IL-6) are known to be able to activate enzyme 11-beta-hydroxysteroid dehydrogenase that interacts in the homeostasis of intracranial pressure. Furthermore, encephalitis, cerebral venous thrombosis, coagulopathy, and hyperviscosity can impair cerebral spinal fluid absorption (106).

Verkuil et al. and Divya et al. observed two different cases of otherwise healthy adolescents affected by MIS-C who developed bilateral papilledema in the absence of any other neurological symptoms and of any lesions in MRI and magnetic resonance venography; increased pressure in cerebrospinal fluid was diagnostic for pseudotumor cerebri (106, 107).

#### 3.2.7. Cranial neuropathy

Involvement of cranial nerves has been rarely reported as a complication of MIS-C. Lonardi et al. described the case of a 2-year-old child who developed, 3 weeks after the resolution of SARS-CoV-2 infection, a monolateral third cranial nerve palsy (exotropia, ptosis, and mydriasis). Cerebrospinal fluid examination documented an albuminocytologic dissociation. The child underwent treatment with oral steroids (108).

## 4. Comparison of neurological manifestations in KD and MIS-C

MIS-C is an emergent SARS-Cov-2-related hyperinflammatory condition that initially seemed to have many similarities to KD, suggesting a common pathogenetic substrate.

Many studies have highlighted the differences from KD, observing that patients affected by MIS-C are usually older, with prominent gastrointestinal manifestations, diffuse adenopathy, extensive conjunctivitis, myocardial damage, and leukopenia and thrombocytopenia at the laboratory examinations (9, 109–113). Moreover, KD has the highest incidence in East Asia and Japan, while MIS-C is more common among non-Hispanic Black children, supporting a possible genetic underlying mechanism (13, 114).

Both conditions can present a neurological involvement as a consequence of cerebral vasculitis caused by immunological hyperactivation, hypoxia, and a state of hypercoagulability (13). While the hypothetical antigen is unclear in KD, the spike protein of SARS-Cov-2 plays an important role in brain injury in MIS-C with a mechanism of molecular mimicry with neuronal gangliosides (18, 79).

The inflammation in KD is caused by a deposit of immune complexes, which induce thrombocytosis and cytokines release likewise. Moreover, it was observed that immunoglobulin A (IgA) is often elevated and may play a role. On the other hand, in MIS-C, the immune activation induced by the spike protein leads to interferon-gamma (IFN-**γ**) and interleukin-1 activation with differentiation 8 (CD8) cytotoxic T cells, causing tissue damage. Both KD and MIS-C show an increase of tumor necrosis factor (TNF), interleukin-1, interleukin-18, interleukin-6, and IFN-**γ** pathways, inducing an amplification of the cytokine activation (115).

Some differences have been noticed between KD and MIS-C in terms of frequency, severity, and clinical expression of their neurological involvement. Neurological involvement seems to be more frequent in patients with MIS-C than in patients with KD: even if its exact prevalence in MIS-C is not yet epidemiologically clearly defined and varies according to different case series, it is on average around 50% while in KD has been described in 1%–30% of patients (116).

Moreover, patients with MIS-C seem to present a more severe clinical pattern at the onset, even if they present a good response to the anti-inflammatory treatment with a consequent good clinical course (20, 109). In patients with KD, on the contrary, the presence of neurological involvement may lead to the risk of misdiagnosis, delayed treatment, and IVIG resistance (20).

Some clinical manifestations, including cerebellitis, delirium, and pseudotumor cerebri, have rarely been described only in patients with MIS-C (86), while both KD and MIS-C can develop aseptic meningoencephalitis (23–30), encephalopathy (59–61, 86, 88, 91–95), cerebral vasculitis, stroke (52–54, 103–105), seizures (62–64, 98), cranial nerve palsy (37–41, 109), and headache (88–90), as reported in Table 3.

**Table 3:** Differences between neurological manifestations of Kawasaki disease and multisystem inflammatory syndrome in children.

Neurological manifestation	Multisystem inflammatory syndrome in children	Kawasaki disease
Aseptic meningoencephalitis	+	+
Subdural effusion	−	+
Cerebral vasculitis and stroke	+	+
Encephalopathy	+	+
Facial nerve palsy	−	+
Abducens nerve palsy	−	+
Oculomotor nerve palsy	+	+
Glossopharyngeal nerve palsy	−	+
Seizures	+	+
Neurodevelopmental disorders	−	+
Sensorineural hearing loss	−	+
Headache	+	+
Cerebellitis	+	−
Delirium	+	−
Pseudotumor cerebri	+	−

The difference between KD and MIS-C may be linked to the different relationship between trigger and immune response in these diseases, which could influence not only the clinical presentation but also the severity, type of damage, and therapeutic response (115).

## 5. Conclusion

The involvement of the nervous system, including neurological and neuropsychiatric disorders, has been described in KD and MIS-C. Epidemiological data on the neurological aspects have been defined in patients with KD; on the contrary, knowledge on MIS-C is limited and needs to be further explored. KD and MIS-C may display a clinical overlap in some neurological manifestations, such as aseptic meningoencephalitis, encephalopathy, cerebral vasculitis, stroke, seizures, and cranial nerve palsy.

Due to the severity of manifestations, an early differential diagnosis is mandatory to start a prompt treatment, reduce the complications, and ameliorate the prognosis of the disease. Further studies on the involvement of the nervous system in patients with MIS-C are needed to clarify additional features, such as long-term comorbidities and NDDs.
